# Monooxygenase, a Novel Beta-Cypermethrin Degrading Enzyme from *Streptomyces* sp

**DOI:** 10.1371/journal.pone.0075450

**Published:** 2013-09-30

**Authors:** Shaohua Chen, Qingsheng Lin, Ying Xiao, Yinyue Deng, Changqing Chang, Guohua Zhong, Meiying Hu, Lian-Hui Zhang

**Affiliations:** 1 College of Natural Resources and Environment, South China Agricultural University, Guangzhou, People’s Republic of China; 2 Institute of Molecular and Cell Biology, Proteos, Singapore, Republic of Singapore; Russian Academy of Sciences, Institute for Biological Instrumentation, Russian Federation

## Abstract

The widely used insecticide beta-cypermethrin has become a public concern because of its environmental contamination and toxic effects on mammals. In this study, a novel beta-cypermethrin degrading enzyme designated as CMO was purified to apparent homogeneity from a *Streptomyces* sp. isolate capable of utilizing beta-cypermethrin as a growth substrate. The native enzyme showed a monomeric structure with a molecular mass of 41 kDa and pI of 5.4. The enzyme exhibited the maximal activity at pH 7.5 and 30°C. It was fairly stable in the pH range from 6.5–8.5 and at temperatures below 10°C. The enzyme activity was significantly stimulated by Fe^2+^, but strongly inhibited by Ag^+^, Al^3+^, and Cu^2+^. The enzyme catalyzed the degradation of beta-cypermethrin to form five products via hydroxylation and diaryl cleavage. A novel beta-cypermethrin detoxification pathway was proposed based on analysis of these products. The purified enzyme was identified as a monooxygenase by matrix-assisted laser desorption/ionization time-of-flight/time-of-flight mass spectrometry analysis (MALDI-TOF-MS) and N-terminal protein sequencing. Given that all the characterized pyrethroid-degrading enzymes are the members of hydrolase family, CMO represents the first pyrethroid-degrading monooxygenase identified from environmental microorganisms. Taken together, our findings depict a novel pyrethroid degradation mechanism and indicate that the purified enzyme may be a promising candidate for detoxification of beta-cypermethrin and environmental protection.

## Introduction

Pyrethroid insecticides have been used worldwide due to their potent toxic activity against various insect pests, and in particular, they have become the dominate insecticides in retail markets [1], [2]. Since 2000, the usage of these pesticides has been increased by as much as 25%, and their application is anticipated to be further increased due to the reduced use of organophosphate insecticides like diazinon and chlorpyrifos [3]. Beta-cypermethrin (beta-CP) [cyano-(3-phenoxyphenyl) methyl 3-(2,2-dichloroethenyl)-2,2-dimethylcyclopropane-1-carboxylate] is one of the most frequently used pyrethroid insecticides. It has been widely used in agriculture, forestry, horticulture, public health, and homes, as well as for protection of textiles and buildings [4], [5].

Persistent and large scale use of beta-CP has resulted in a serious environmental contamination problem, and which poses a serious threat to the health of human beings and ecosystems [6]–[10]. For instance, the pesticide has been detected in nearly all the urban creeks in California [3], [11]–[13]. Beta-CP is highly toxic to fish and aquatic invertebrates [8], [14]–[16]. Moreover, it shows carcinogenic and endocrine disrupting activities on mammals [6], [17]–[19]. Furthermore, the pesticide is known to adversely affect reproductive function, and the development of sexual and nervous systems [20]–[24]. The findings press the critical need for developing effective and economic approaches to remove this contaminate from environments.

Biodegradation has been attracting much attention in cleanup of the contaminated environments because conventional physical and chemical methods for disposal of persistent pollutants are low in efficiency and need comparatively high operating cost [25]–[30]. Several beta-CP degradation mechanisms have been identified in recent years, such as beta-CP degrading bacterial isolates *Serratia* sp. JCN13 [31]; *Ochrobactrum lupini* DG-S-01 [32], and *Pseudomonas aeruginosa* CH7 [5], and the three genes, i.e., *Estp*, *pytH*, and *PytZ,* encoding pyrethroid-degrading hydrolases from *Klebsiella* sp. ZD112, *Sphingobium* sp. JZ-1, and *Ochrobactrum anthropi* YZ-1, respectively [33]–[35]. In addition, one thermostable enzyme Sys410 involved in pyrethroid degradation has recently been isolated from Tuban Basin soil using metagenomic approach [36]. However, little information is available regarding the ability of actinomycetes in degradation of beta-CP. In this study, we described the purification and characterization of a novel beta-CP degrading enzyme from the actinomycete *Streptomyces* sp., previously isolated from the pyrethroid-contaminated soils [4]. The purpose of this study was to investigate its specific role in beta-CP degradation. To the best of our knowledge, this is the first pyrethroid-degrading enzyme purified to homogeneity from actinomycetes.

## Materials and Methods

### Chemicals

Beta-CP (94.8% purity) was obtained from Zhongshan Aestar Fine Chemical Inc., Ltd, China. Sephacryl™ S-100 (16/60) and diethylaminoethyl cellulose (DEAE) were purchased from General Electric Company, USA. Chromatographic grade acetonitrile were purchased from Sigma-Aldrich, USA. Sodium dodecyl sulfate (SDS) and polyacrylamide were purchased from Amresco, USA. All the other chemicals and solvents used in this study were at analytical grade.

### Microorganisms and Culture Conditions

The mineral salt medium (MSM) containing (g·L^−1^) (NH_4_)_2_SO_4_, 2; MgSO_4_·7 H_2_O, 0.2; CaCl_2_·2 H_2_O, 0.01; FeSO_4_·7 H_2_O, 0.001, Na_2_HPO_4_·12 H_2_O, 1.5; and KH_2_PO_4_, 1.5 was used for the cultivation of pyrethroid-degrading strains. The actinomycete isolate, which was used in this study, was isolated from the pyrethroid-contaminated soils using an enrichment culture technique [4].

For enzyme production, fresh MSM medium supplemented with 50 mg·L^−1^ of beta-CP was inoculated with *Streptomyces* sp. viable spores. The culture was incubated at 28°C for 5 days in 500 mL-Erlenmeyer flasks containing 200 mL of medium on a rotary shaker at 150 rpm, harvested by centrifugation at 8000×*g* for 30 min at 4°C, washed twice with cold phosphate buffer solution (PBS, 50 mM, pH 7.5), and stored at −20°C until further use.

### Preparation of Crude Cell Extracts

To prepare crude cell extracts, the harvested mycelia were re-suspended in 50 mM PBS buffer (pH 7.5) to a final concentration of 25 g cell dry weight per litre. The mycelia cells were disrupted in an ultrasonic cell disruption system at 4°C. The crude cell extracts were obtained after removing cellular debris by centrifugation at 12,000×*g* for 30 min at 4°C. The resulting supernatants were used as an enzyme source for further purification. Protein concentration was determined by the method of Bradford [37] with bovine serum albumin as a standard.

### Enzyme Assays

Pesticide catalytic activity was assayed by adding 0.05 mL enzyme solution to 5 mL of 50 mM PBS (pH 7.5) containing 5 mg·L^−1^ of beta-CP and incubating for 10 min at 30°C. After that, samples were extracted as described below, and the residues of pesticide were determined by high performance liquid chromatography (HPLC) according to the method described by Chen et al. [38]. Every treatment was performed in triplicate with inactivated enzyme as control. One unit of enzyme activity (U) was defined as the amount required to catalyze formation of 1 µmol of product per minute.

### Enzyme Purification

All of the experiments below were carried out at 4°C unless otherwise specified. Purification was performed according to the methods described in previous reports [39]–[41] with minor modifications.

- Ammonium sulfate precipitation. The crude cell extracts was brought to 55% ammonium sulfate saturation and stirred for 30 min, the cloudy suspension was centrifuged at 12,000×*g* for 20 min, and the supernatant was brought to 60% ammonium sulfate saturation; the pellet obtained by centrifugation was dissolved in the smallest possible volume of 50 mM PBS (pH 7.5) and dialyzed 1000-fold against 50 mM PBS (pH 7.5), and the supernatant was finally concentrated to 2 mL.
- DEAE-Sepharose ion exchange chromatography. The dialyzed enzyme solution after ammonium sulfate saturation was loaded onto DEAE-Sepharose Fast Flow anion-exchange column (2.5×30 cm) that had been pre-equilibrated with 50 mM PBS (pH 7.5). The column was washed with 20 mL of the same buffer, and proteins were eluted with a linear gradient of NaCl solution in the range of 0 to 1.0 M in the equilibrating buffer. Fractions (5 mL) were collected every 5 min and screened for enzyme activity. Active fractions were pooled for further purification.
- Sephacryl™ S-100 (16/60) gel filtration. The concentrated enzyme solution after DEAE-Sepharose Fast Flow anion-exchange was further purified by a Sephacryl™ S-100 column (2.5×100 cm). The column was washed at a flow rate of 0.5 mL·min^−1^ with 180 mL of 50 mM PBS buffer (pH 7.5), and 5-mL fractions were collected. The fractions with high specific activity were then pooled and concentrated for subsequent studies.

### Determination of Molecular Mass and pI

The molecular mass of the denatured protein was determined by sodium dodecyl sulfate-polyacrylamide gel electrophoresis (SDS-PAGE). An SDS-12.5% polyacrylamide gel was prepared according to the method of Laemmli [42]. Proteins were stained with Coomassie brilliant blue R-250. The molecular mass of the native protein was estimated by gel filtration on the Sephacryl™ S-100 (16/60). Ovalbumin (43.0 kDa), carbonic anhydrase (31.0 kDa), and lysozyme (14.4 kDa) were used as the standard proteins. Isoelectric point (pI) was determined by isoelectric focusing calibration [39]–[41].

### Effects of Temperature and pH on Activity and Stability of the Enzyme

To determine the optimal temperature and pH, the enzyme catalytic activity was investigated by incubating enzyme (0.05 mL) with 5 mg·L^−1^ beta-CP as a substrate for 10 min in 50 mM PBS. At pH 7.5, catalytic activity was determined in various temperatures ranging from 18°C to 40°C. At temperature 30°C, catalytic activity was assayed at pH values ranging from 4.0 to 11.0 [34], [41]. The relative residual activity was measured immediately as described previously. Here, the relative catalytic activity of the pre-incubated sample at 30°C was regarded as 100%.

Thermostability was measured by pre-incubation of the enzyme for different times in 50 mM PBS (pH 7.5) at different temperatures. The pH stability was tested after incubation of the enzyme for 2 h at 30°C [36]. The retaining catalytic activity of the enzyme was determined as mentioned above.

### Effects of Different Metal Ions on Enzyme Catalytic Activity

For determination of the effects of different metal ions on catalytic activity, enzyme assay was performed in 50 mM PBS (pH 7.5) with 5 mg·L^−1^ beta-CP as a substrate and with various metal ions at a final concentration of 10 mM. The activity assayed in the absence of metal ions was defined as control [35]. The metal ions tested including Fe^2+^, Mg^2+^, Ca^2+^, K^+^, Na^+^, Zn^2+^, Ba^2+^, Mn^2+^, Cu^2+^, Al^3+^, and Ag^+^.

### NH_2_-terminal Amino Acid Sequencing

The purified enzyme was electrophoretically transferred from a 12.5% SDS-PAGE to a polyvinylidene fluoride (PDVF) membrane (Millipore, USA). The region containing the beta-CP catalysis band was cut out, and the protein was sent to Shanghai GeneCore BioTechnologies Co., Ltd., China, for NH_2_-terminal amino acid sequencing [40], [41]. Sequencing was performed by matrix-assisted laser desorption/ionization time-of-flight/time-of-flight mass spectrometer (MALDI-TOF-MS; Bremen, Germany) according to the method of Peng et al. [43].

### Identification of the Beta-CP Transformation Products

The beta-CP transformation products in the reaction solution were collected at different catalytic times. After acidification to pH 2 with 2 M HCl, the reaction solution was extracted according to the method described in a previous report [44]. The products identified by mass spectrometry analysis were matched with authentic standard compounds from the National Institute of Standards and Technology (NIST, USA) library database.

The transformation products of beta-CP by the enzyme were identified on an Agilent 6890N/5975 gas chromatopraphy-mass spectrometry (GC-MS) system equipped with auto-sampler, an on-column, split/splitless capillary injection system, and with HP-5MS capillary column (30.0 m×250 µm×0.25 µm) with array detection from 30–500 nm (total scan). Helium was used as a carrier gas at a flow rate of 1.5 mL·min^−1^. The column was held initially at a temperature of 90°C for 2 min, then at 6°C·min^−1^ to 150°C for 1 min, at 10°C·min^−1^ to 180°C for 4 min, and finally at 20°C·min^−1^ to 260°C for 10 min. The temperatures corresponding to transfer line and the ion trap were 280°C and 230°C, respectively. The column outlet was inserted directly into the electron ionization source block, operating at 70 eV. The injection volume was 1.0 µL with splitless sampling at 250°C [45], [46].

## Results

### Production of the Enzyme

*Streptomyces* sp. was aerobically cultured in the MSM medium supplemented with beta-CP as a growth substrate. Beta-CP degrading enzyme activity was detected at the late log phase and reached the maximum level after 5 days of cultivation (Fig. S1). However, when the pesticide in the MSM medium was replaced by an equal amount of sucrose or glucose, the crude cell extracts showed no or only a trace level of the enzyme activity. These results suggest that the enzyme was induced by beta-CP in the medium. This observation is reminiscent of a previous finding that the pyrethroid hydrolase from *Aspergillus niger* ZD11 was inducible by *trans*-permethrin [39].

### Purification and the Molecular Mass of the Enzyme

**Figure 1:**
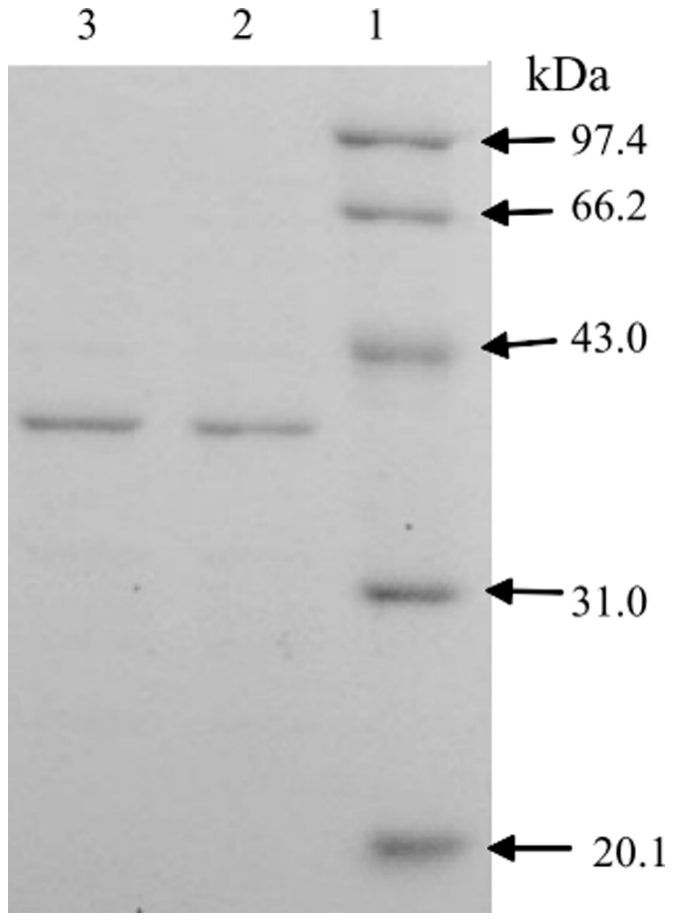
SDS-PAGE analysis of the purified enzyme from *Streptomyces* sp. (Lane 2 and 3) and protein markers (Lane 1) stained with Coomassie blue. Markers from top to bottom are phosphorylase b (97.4 kDa), bovine serum albumin (66.2 kDa), ovalbumin (43.0 kDa), carbonic anhydrase (31.0 kDa), and trypsin inhibitor (20.1 kDa).

The beta-CP degrading enzyme was purified from the crude cell extracts of *Streptomyces* sp. by ammonium sulfate precipitation, DEAE-Sepharose Fast Flow anion-exchange chromatography, and Sephacryl™ S-100 gel filtration chromatography. The results of purification were summarized in Table 1. The enzyme was purified by 26.1-fold to a specific activity of 21.4 U·mg^−1^ protein from the cells with a yield of 21.2%. The purified enzyme gave a single band in SDS-PAGE, suggesting the purified sample was electrophoretically homogeneous under the dissociating conditions. The molecular mass of the purified enzyme determined by SDS-PAGE analysis was approximately 41 kDa (Fig. 1). SDS-PAGE analysis of the fractions showing beta-CP degrading enzyme activity obtained during the purification was given in Fig. S2. The relative molecular mass of native enzyme estimated by gel filtration on a calibrated column of Sephacryl™ S-100 was around 41 kDa. These results suggest that the purified enzyme is a monomer. The pI value was determined to be 5.4. Herewith, we designated the purified enzyme as CMO for the convenience of description.

**Table 1:** Purification of beta-CP degrading enzyme from *Streptomyces* sp.

Purification steps	Total protein (mg)	Total activity (U)^a^	Specific activity (U·mg^−1^)	Yield (%)	Purification fold
Crude cell extracts	1702.4	1674.8	1.0	100	1.0
Ammonium sulfate precipitation	108.6	599.0	5.5	35.8	5.6
DEAE-Sepharose Fast Flowanion-exchange chromatography	36.3	382.8	10.5	22.9	10.7
Sephacryl™ S-100 gel filtration	16.6	355.7	21.4	21.2	26.1

Note: ^a^One unit of enzyme activity (U) was defined as the amount required to catalyze formation of 1 µmol of product per minute.

### Effect of Temperature on CMO Activity and Stability

**Figure 2:**
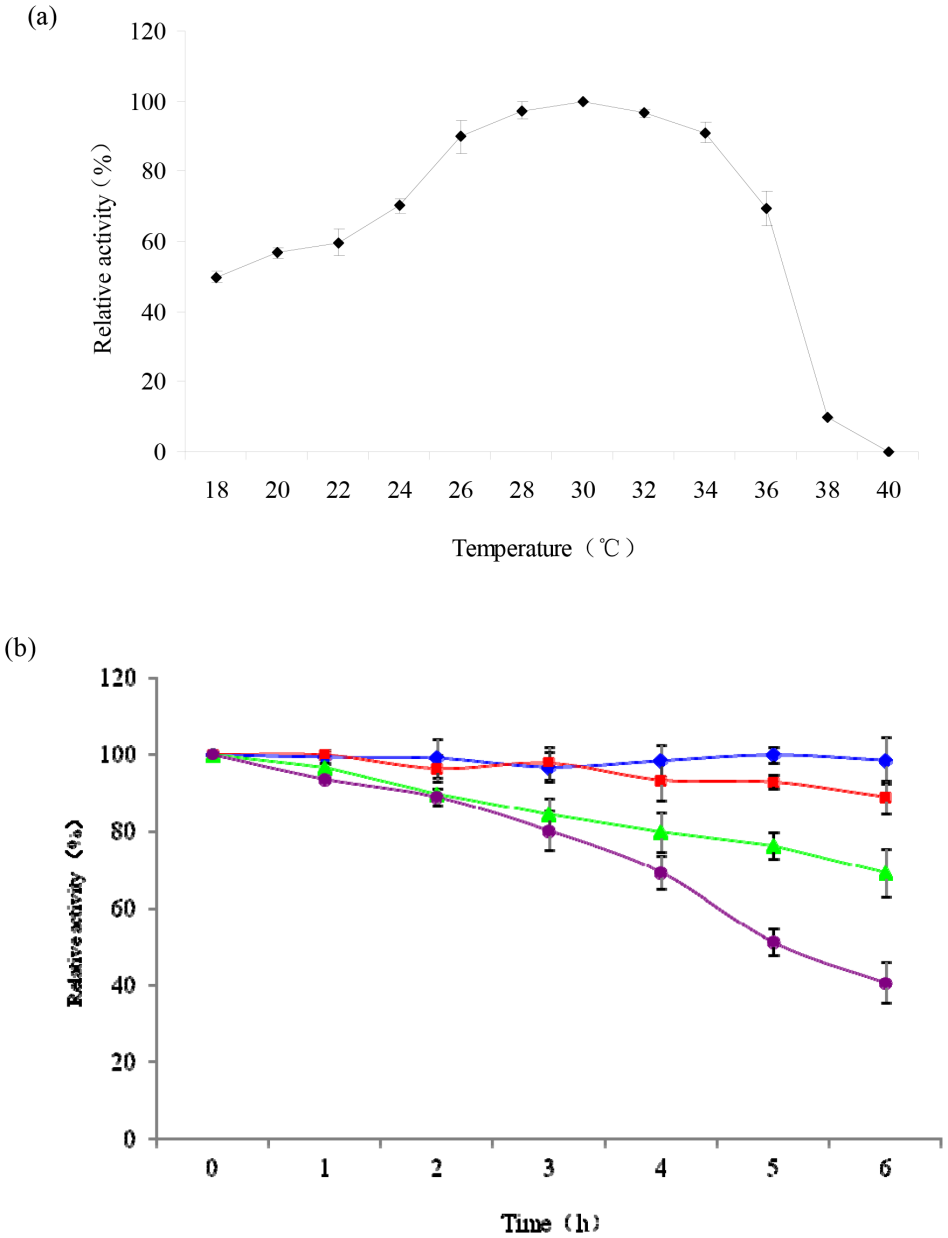
Effect of temperature on enzyme activity (a) and stability (b) of CMO. The purified enzyme was pre-incubated at 0°C (♦), 10°C (▪), 20°C (▴), and 30°C (•) for 6 h. Error bar represents the standard deviation of the mean of three replicates.

The effect of temperature on CMO activity was investigated by using beta-CP as a substrate at pH 7.5, with temperature ranging from 18°C to 40°C. Catalytic activity was increased as temperature increased up to 30°C, and decreased beyond that level. CMO displayed a high catalytic activity at temperatures between 25°C and 35°C with an optimal temperature at 30°C (Fig. 2a).

Thermostability of the purified enzyme was determined by analysis of residual activity at regular intervals after pre-incubation for 6 h, at temperatures ranging from 0 to 30°C. CMO was highly stable below 10°C, with a residual activity exceeding 85% after incubation for 6 h. At temperatures above 10°C, catalytic activity was decreased slowly along with increased incubation time. The enzyme retained approximately 60% and 50% of its activity after incubation for 6 h at 20°C and 30°C, respectively (Fig. 2b).

### Effect of pH on CMO Activity and Stability

**Figure 3:**
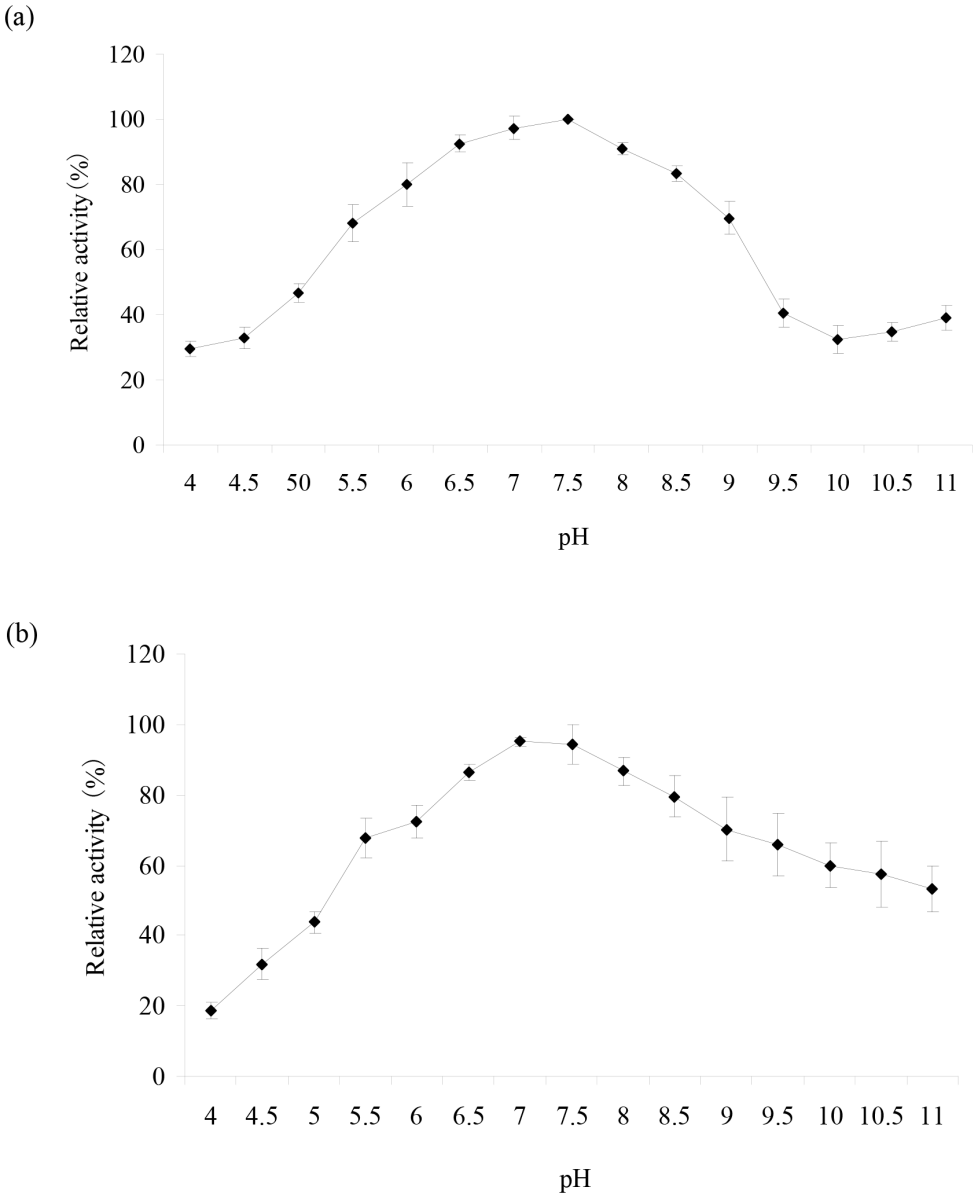
Effect of pH on enzyme activity (a) and stability (b) of CMO. Error bar represents the standard deviation of the mean of three replicates.

The effect of pH on CMO activity was determined using beta-CP as a substrate at 30°C, with pH values ranging from 4.0 to 11.0. Maximal activity was observed at pH 7.5. The enzyme showed high activity in a broad pH range with 80% of residual activity between pH values 6.5 and 8.5 (Fig. 3a).

pH stability was measured via 2 h pre-incubation of the purified enzyme in the above buffer at different pH values ranging from 4.0 to 11.0 at 30°C. The results in Fig. 3b show that the enzyme was fairly stable at pH values between 6.5 to 8.5, retaining more than 80% of the original activity after pre-incubation for 2 h. However, at pH values of 4.0 and 11.0, CMO only kept about 20% and 40% of its activity after incubation for 2 h, respectively. These results suggest that the purified enzyme is more sensitive to acidic conditions than alkaline conditions.

### Effect of Metal Ions on CMO Activity

The influence of various metal ions on the enzyme activity of CMO was investigated by addition of metal ions separately into the reaction mixture at a final concentration of 1.0 mM. The CMO activity was then measured with beta-CP as a substrate and expressed as a percentage of the activity obtained in the absence of the added compounds (Table 2). Ag^+^, Al^3+^, and Cu^2+^ had a strong inhibitory effect (40–50% inhibition); while Zn^2+^, Mn^2+^, and Ba^2+^ showed only slight inhibitory effect (5–10% inhibition), respectively. Other metal ions such as Na^+^ and K^+^ had no obvious effect on the enzyme activity. In contrast, the presence of Ca^2+^ and Mg^2+^ resulted in slight stimulation (10–20% stimulation) of the enzymatic activity. In particular, the CMO activity was drastically boosted by Fe^2+^ up to 317.2%, suggesting that Fe^2+^ may be associated with the catalytic center of the enzyme.

**Table 2:** Effects of various metal ions on beta-CP degrading enzyme activity.

Metal ions	Relative activity (%)	Metal ions	Relative activity (%)
Fe^2+^	317.2±16.3a	Ba^2+^	98.9±14.7b
Mg^2+^	109.7±13.9b	Mn^2+^	94.8±8.4b
Ca^2+^	117.4±11.0b	Cu^2+^	62.2±9.2c
K^+^	106.3±14.9b	Al^3+^	58.6±7.4c
Na^+^	101.1±8.6b	Ag^+^	51.5±10.0c
Zn^2+^	89.4±10.6b		

Note: The data presented are means of three replicates with standard deviation, which is within 5% of the mean.

### Protein Identification

**Figure 4:**
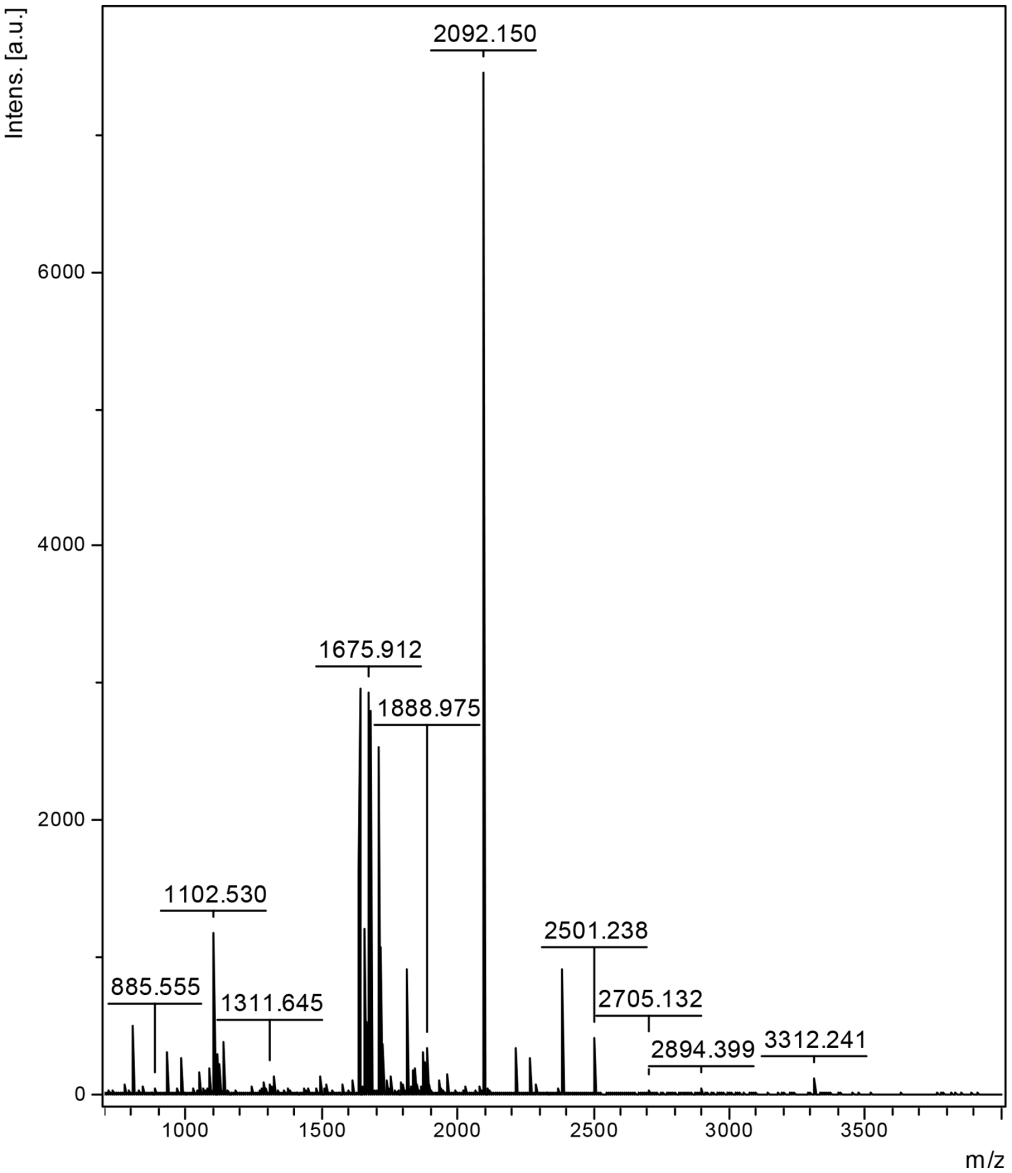
Matrix-assisted laser desorption/ionization time-of-flight/time-of-flight mass spectrometry analysis (MALDI-TOF-MS) of the purified enzyme.

The purified protein was identified by matrix-assisted laser desorption/ionization time-of-flight/time-of-flight mass spectrometry analysis (MALDI-TOF-MS) and N-terminal protein sequencing (Fig. 4). Database searches showed that the protein matched well with the monooxygenase from *Streptomyces* genus.

### Identification of the Beta-CP Transformation Products

**Figure 5:**
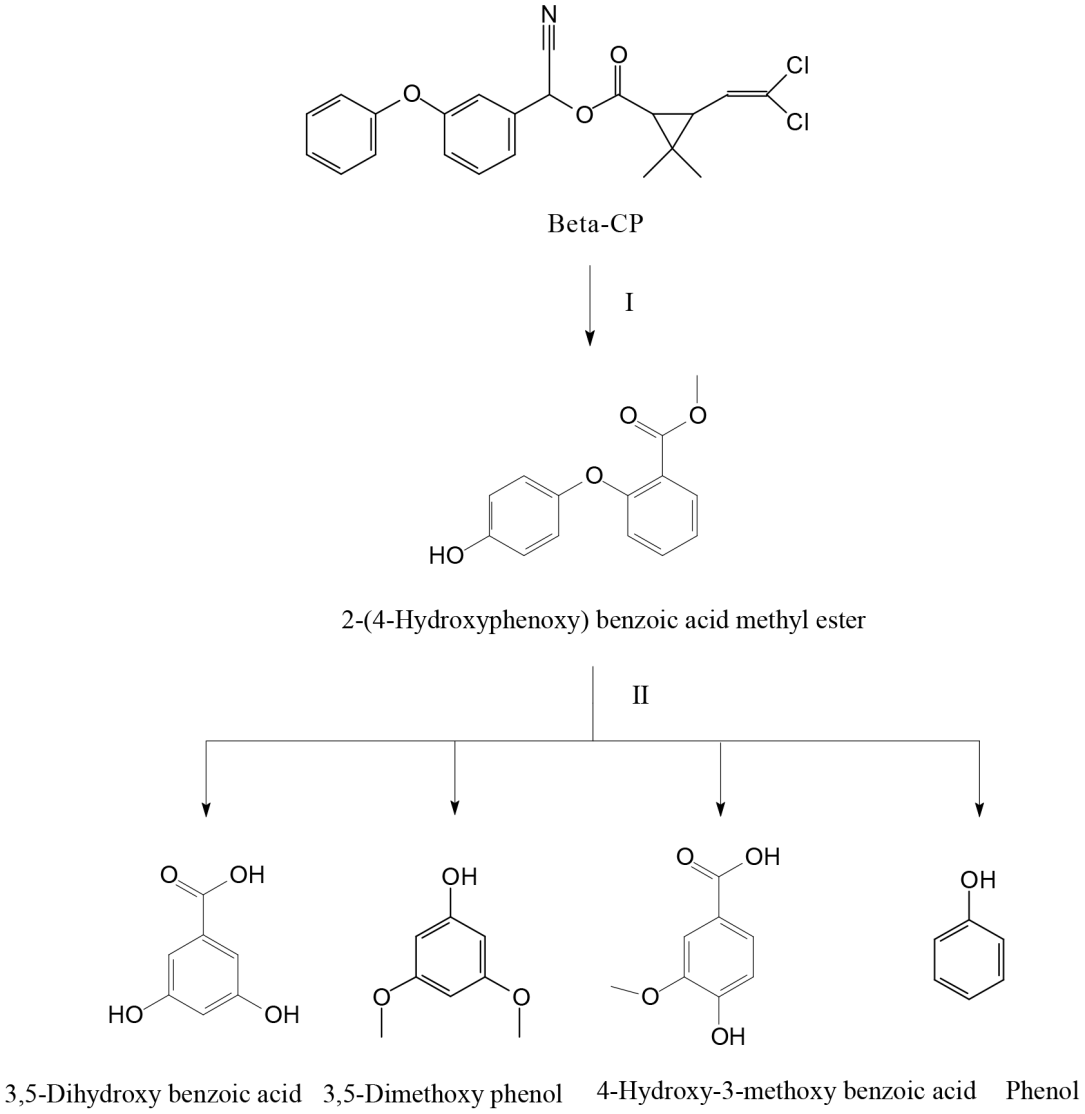
The proposed pathway for detoxification of beta-CP by the enzyme from *Streptomyces* sp. I, hydroxylation; II, diaryl cleavage.

The enzyme catalyzed the degradation of beta-CP to five products in the PBS buffer, including 2-(4-hydroxyphenoxy) benzoic acid methyl ester, 3,5-dihydroxy benzoic acid, 3,5-dimethoxy phenol, 4-hydroxy-3-methoxy benzoic acid, and phenol. The transformation products were identified and confirmed by GC-MS, based on the similarity of their fragment and molecular ions with those of corresponding authentic compounds (Fig. S3). The retention time (RT) of these compounds was presented in Table 3. On the basis of the transformation products formed, a novel detoxification pathway for beta-CP was proposed (Fig. 5). The monooxygenase first converted the parent beta-CP [A] to 2-(4-hydroxyphenoxy) benzoic acid methyl ester [B] via hydroxylation. The intermediate was then subjected to diaryl cleavage, resulting in formation of 3,5-dihydroxy benzoic acid [C], 3,5-dimethoxy phenol [D], 4-hydroxy-3-methoxy benzoic acid [E], and phenol [F]. This is the first evidence of a novel beta-CP detoxification mechanism by hydroxylation and diaryl cleavage, which we propose is of vital importance in the beta-CP biogeocycle.

**Table 3:** Chromatographic properties of the beta-CP degradation products by the enzyme from *Streptomyces* sp.

Code	RT (min)	*m*/*z*	Compounds
A_1_	31.145	415	*trans*-Beta-CP
A_2_	30.961	459	*cis*-Beta-CP
B	15.903	497	2-(4-Hydroxyphenoxy) benzoic acid methyl ester
C	9.650	429	3,5-Dihydroxy benzoic acid
D	9.576	460	3,5-Dimethoxy phenol
E	8.046	232	4-Hydroxy-3-methoxy benzoic acid
F	3.653	281	Phenol

## Discussion

In this study, a novel pyrethroid-degrading enzyme designated as CMO was purified to apparent homogeneity from a *Streptomyces* sp. isolate capable of utilizing beta-CP as a growth substrate. The pyrethroid-degrading enzyme activities have been reported from several bacterial and fungal isolates [33], [34], [35], [39], [40], [47], but not from actinomycetes. This is the first report to our knowledge on the production, purification, and characterization of a pyrethroid-degrading enzyme from actinomycetes.

CMO has an apparent molecular mass of 41 kDa, which is smaller than the reported pyrethroid-hydrolyzing enzymes, such as permethrinase (61 kDa) from *Bacillus cereus* SM3 [47], pyrethroid hydrolase (56 kDa) from *A. niger* ZD11 [39], and EstP (73 kDa) from *Klebsiella* sp. ZD112 [33]. However, it was bigger than esterase (31.2 kDa) from the metagenome [48], pytH (31 kDa) from *Sphingobium* sp. JZ-1 [34], PytZ (24.2 kDa) from *Ochrobactrum anthropi* YZ-1 [35], and Sys410 (30.8 kDa) from the metagenome [36]. The pI value of CMO was estimated to be 5.4, which was similar to the value of the pyrethroid hydrolase from *A. niger* ZD11 (5.4), but lower than that recorded for *Klebsiella* sp. ZD112 (8.6) [33], [39]. The low pI suggests that the enzyme is rich in acidic amino acid residues [40], [41]. Taken together, the above findings suggest that CMO could be a novel pyrethroid-degradation enzyme.

CMO displayed a high catalytic activity at temperatures between 25°C and 35°C with an optimal temperature at 30°C. This result was in consistent with the observation that the optimal temperature for pyrethroid-degrading enzymes is usually between 30°C and 45°C [33], [35], [39], [40], [47], [48]. However, it was far less than that recorded for the thermostable pyrethroid-hydrolyzing enzyme Sys410 (55°C) [36].

CMO showed high activity in a broad pH range between pH values 6.5 and 8.5. Maximal activity was observed at pH 7.5. A range of previous characterized pyrethroid-degrading enzymes show maximal activity at pH 7.5 [35], [40], [47], but the pyrethroid esterase from various bacterial isolates or metagenome are known to exhibit maximal activity at pH range from 6.5–7.0 [33], [36], [39], [48]. Similar results were observed in most other pyrethroid-degrading enzymes [33], [34], [35], [39], [40]. Notably, CMO retained a high activity at a broad range of temperatures and pH, suggesting the promising potential of using this enzyme to clean up beta-CP contamination under various environmental conditions.

The influence of various metal ions on the enzyme activity of CMO was investigated. The enzyme activity was significantly stimulated by Fe^2+^. This result contrasts with previous findings of Zhai et al. [35] who reported that Fe^2+^ had no significant effect on the pyrethroid-hydrolyzing carboxylesterase (PytZ) isolated from *O. anthropi* YZ-1. Our results suggested that Fe^2+^ may be involved in the catalytic center of the enzyme. However, the CMO activity was strongly inhibited by Ag^+^, Al^3+^, and Cu^2+^. Similar results were observed in other pyrethroid-degrading enzymes isolated from *A.* *niger* ZD11, *Klebsiella* sp. ZD112, *Sphingobium* sp. JZ-2, and *O.* *anthropi* YZ-1 [33], [35], [39], [40].

The purified enzyme was identified as a monooxygenase by matrix-assisted laser desorption/ionization time-of-flight/time-of-flight mass spectrometry analysis (MALDI-TOF-MS) and N-terminal protein sequencing. Until now, all the reported pyrethroid-degrading enzymes from environmental isolates belong to hydrolase family [33], [34], [35], [39], [40], [47], whereas our results suggest that the purified enzyme is most likely to be a monooxygenase.

Generally, carboxylic ester hydrolysis by carboxylesterases is considered as the first step of degradation and detoxification of pyrethroid insecticides in many species, from mammals and insects to bacteria [5], [26], [49], [50]. In this study, the transient accumulation of intermediate 2-(4-hydroxyphenoxy) benzoic acid methyl ester suggests that transformation of beta-CP by CMO was initiated by hydroxylation (Fig. 5, step I), which is differing from other pyrethroid hydrolases involved in pyrethroid degradation through hydrolysis [33], [34], [35], [39], [40]. Moreover, the enzyme further transformed the intermediate 2-(4-hydroxyphenoxy) benzoic acid methyl ester by cleavage of the diaryl bond (Fig. 5, step II), leading to detoxification of beta-CP. This is a sharp contrast to the reported pyrethroid hydrolases, which are known to undertake one-step reaction only (hydrolysis) [39], [40]. Given that the CMO enzyme is capable of complete degradation of beta-CP to generate simple aromatic compounds, the enzyme may be an ideal candidate for bioremediation of the beta-CP contaminated environments.

In conclusion, a novel beta-CP degrading enzyme was successfully purified from *Streptomyces* sp. in the present study. The strong catalytic activity and broad temperature and pH range of the enzyme were necessary to meet the practical requirements of bioremediation to enable its use *in situ* for detoxification of beta-CP. Moreover, we presented evidence that the enzyme harbors the catalytic mechanisms for complete degradation of beta-CP to yield simple aromatic products. To our knowledge, this is the first report that a pyrethroid-degrading enzyme transforms beta-CP through hydroxylation and diaryl cleavage, which we propose is of vital importance in beta-CP biogeocycle. Finally, this is the first pyrethroid-degrading monooxygenase purified to homogeneity from environmental isolates.

## Supporting Information

Figure S1 The crude enzyme activity on degrading beta-CP over the incubation time.
(TIF)

Figure S2 SDS-PAGE analysis of the fractions showing beta-CP degrading enzyme activity obtained during the purification. Lane M: protein markers, 14.4–94.0 kDa; Lane 1: total proteins; Lane 2: fractions from ammonium sulfate precipitation; Lane 3: fractions from DEAE Sepharose Fast Flow anion-exchange.
(TIF)

Figure S3 GC-MS spectra of products produced from beta-CP degradation by CMO. a, *trans*-Beta-CP; b, *cis*-Beta-CP; c, 2-(4-Hydroxyphenoxy) benzoic acid methyl ester; d, 3,5-Dihydroxy benzoic acid; e, 3,5-Dimethoxy phenol; f, 4-Hydroxy-3-methoxy benzoic acid; g, Phenol.
(TIF)
