# Supplementary figures and images for: Monooxygenase, a Novel Beta-Cypermethrin Degrading Enzyme from Streptomyces sp
Source: PLoS One. 2013 Sep 30;8(9):e75450. doi: 10.1371/journal.pone.0075450 (PMC3787105; doi:10.1371/journal.pone.0075450)

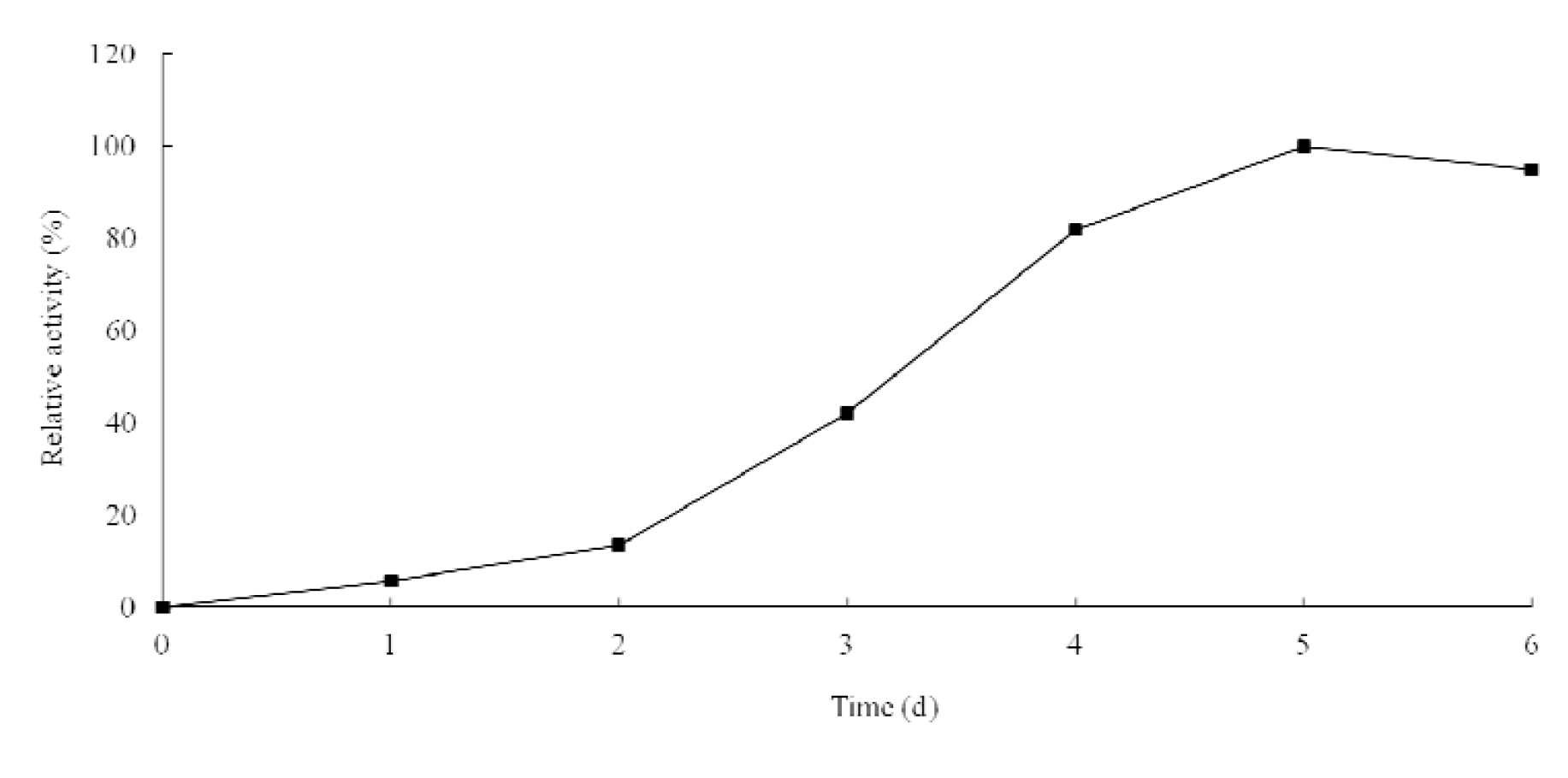

Supplement: Figure S1 — The crude enzyme activity on degrading beta-CP over the incubation time. (TIF) [file pone.0075450.s001.tif]

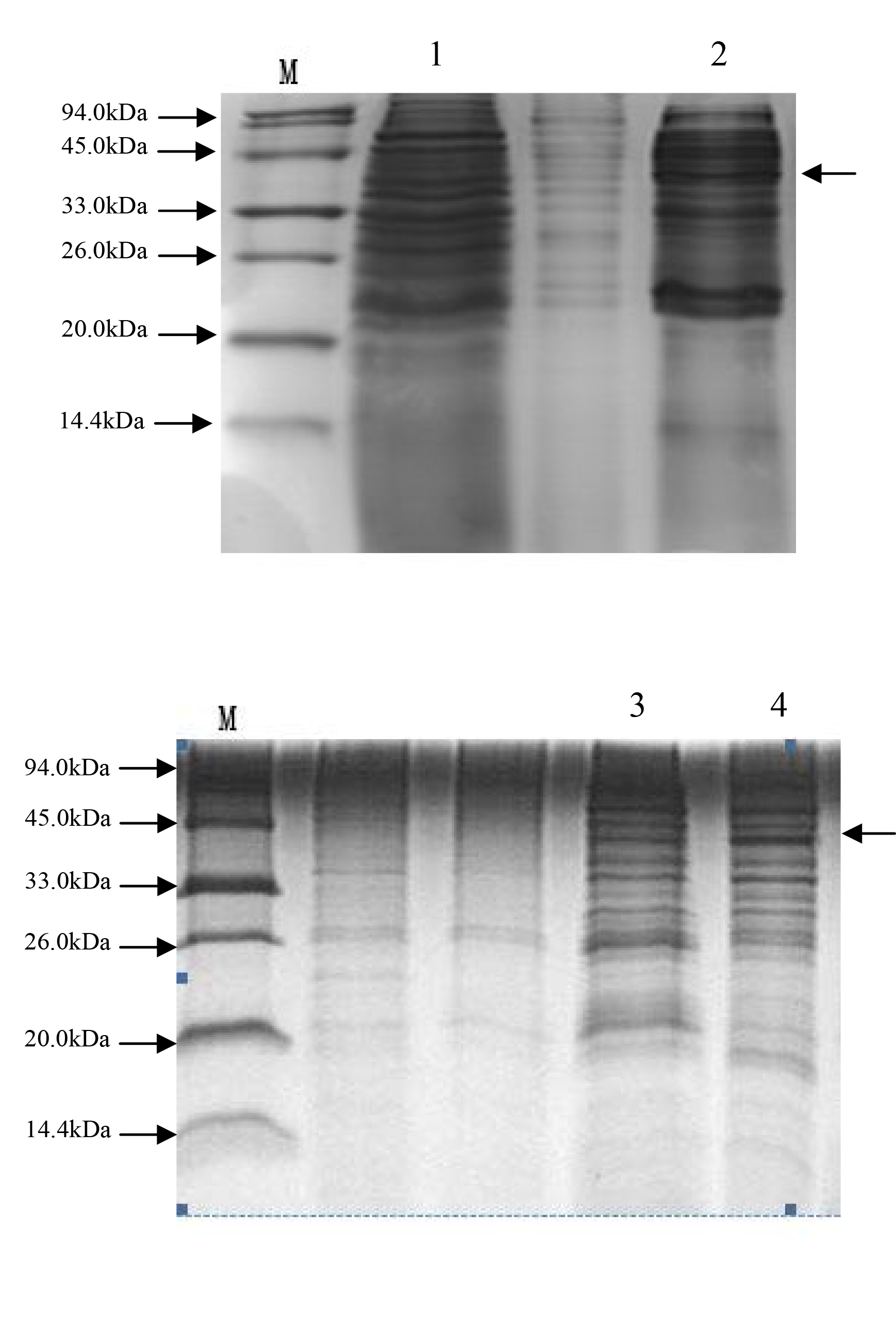

Supplement: Figure S2 — SDS-PAGE analysis of the fractions showing beta-CP degrading enzyme activity obtained during the purification. Lane M: protein markers, 14.4–94.0 kDa; Lane 1: total proteins; Lane 2: fractions from ammonium sulfate precipitation; Lane 3: fractions from DEAE Sepharose Fast Flow anion-exchange. (TIF) [file pone.0075450.s002.tif]

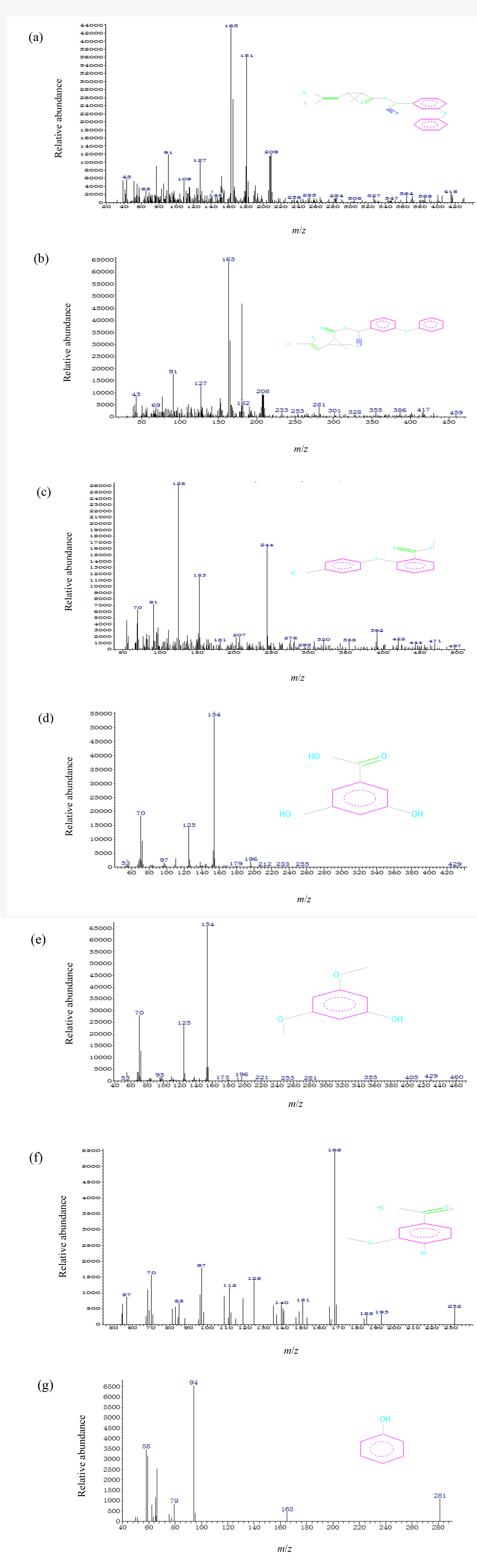

Supplement: Figure S3 — GC-MS spectra of products produced from beta-CP degradation by CMO. a, trans-Beta-CP; b, cis-Beta-CP; c, 2-(4-Hydroxyphenoxy) benzoic acid methyl ester; d, 3,5-Dihydroxy benzoic acid; e, 3,5-Dimethoxy phenol; f, 4-Hydroxy-3-methoxy benzoic acid; g, Phenol. (TIF) [file pone.0075450.s003.tif]
